# The BosR Is Back!

**DOI:** 10.1111/mmi.70046

**Published:** 2026-01-02

**Authors:** D. Scott Samuels, Meghan C. Lybecker

**Affiliations:** ^1^ Division of Biological and Biomedical Sciences University of Montana Missoula Montana USA; ^2^ Bacterial Diseases Branch, Division of Vector‐Borne Diseases National Center for Emerging and Zoonotic Infectious Diseases, Centers for Disease Control and Prevention Fort Collins Colorado USA

**Keywords:** *Borrelia* (*Borreliella*) *burgdorferi*, DNA binding, ferric uptake regulator (FUR), gene regulation, RNA binding

## Abstract

BosR is a novel nucleic acid‐binding protein in the ferric uptake regulator (FUR) family that regulates gene expression in the Lyme disease spirochete *Borrelia* (*Borreliella*) *burgdorferi*. This issue of *Molecular Microbiology* contains a comprehensive transcriptomic study that keenly defines the regulatory swath of BosR in the vertebrate host of *B. burgdorferi* . Despite homology to Fur‐like and PurR‐like orthologs, BosR has traditionally been linked to regulation of RpoS, the alternative sigma factor that controls the regulon required for establishing a vertebrate infection. However, BosR regulates other genes through an RpoS‐independent mechanism, which is elegantly elaborated in Grassmann et al., along with clearly demonstrating that BosR does not participate in the defense against oxidative and nitrosative stress in the vertebrate. However, the recently recognized role of BosR as an RNA‐binding protein with RNA chaperone activity that regulates gene expression in a post‐transcriptional fashion is not wholly appreciated, which clouds the results on determining the DNA‐binding site in vivo. Regardless, this seminal study enshrines BosR as a major regulator of gene expression in *B. burgdorferi* and delineates a multitude of BosR‐regulated cellular functions in the spirochete related to its ability to navigate between its tick vector and vertebrate host in nature as well as to persist in these two disparate environments.

The spirochete *Borrelia* (*Borreliella*) *burgdorferi* is an etiologic agent of Lyme disease (Benach et al. 1983; Burgdorfer et al. 1982; Steere et al. 1983). The spirochete subsists in an enzootic cycle that entails a vertebrate host and a tick vector (Caimano et al. 2016; Radolf et al. 2012; Telford 3rd and Goethert 2021). *B. burgdorferi* wields three major gene regulatory systems to adapt to its environment and persist in each phase of its enzootic cycle: the RpoN‐RpoS alternative sigma (*σ*) factor cascade, the Hk1/Rrp1 two‐component system and its product c‐di‐GMP, and the stringent response (George and Ouyang 2025; Samuels et al. 2021; Stevenson 2023). In addition, several DNA‐binding and RNA‐binding proteins have been implicated; one of the first identified and perhaps best characterized is BosR.

BosR (evidently erroneously *Borrelia* oxidative stress regulator) is a member of the ferric uptake regulator (FUR) family (Boylan et al. 2003; Fraser et al. 1997; Katona et al. 2004; Samuels and Radolf 2009; Seshu et al. 2004; Steingard and Helmann 2023). *B. burgdorferi* does not rely on iron (Posey and Gherardini 2000; Wang et al. 2012), so BosR seemed unlikely to regulate iron metabolism; instead, BosR is analogous to PerR and was first reported to regulate the expression of *dps*/*napA*/*bicA* as well as other genes involved in oxidative stress response in *B. burgdorferi* (Boylan et al. 2003). However, as first established in two studies 17 years ago in this journal (Hyde et al. 2009; Ouyang et al. 2009), and highlighted in a MicroCommentary (Samuels and Radolf 2009), BosR was shown to activate *rpoS* transcription and, thus, the RpoS‐dependent regulon that controls the vertebrate phase of the enzootic cycle, as well as repress genes involved in the tick phase of the enzootic cycle (Grassmann et al. 2023; Hyde et al. 2009, 2010; Katona 2015; Ouyang et al. 2011, 2009, 2014; Shi et al. 2014; Wang et al. 2013).

Considerable gaps remained in our understanding of BosR function, partly because much of the work was done either in a test tube or in cultured spirochetes. Biochemical studies defined DNA‐binding sites in vitro (Katona 2015; Ouyang et al. 2011, 2014) and transcriptomic studies described the BosR‐dependent regulon (Hyde et al. 2006; Ouyang et al. 2009), but there are significant caveats with both approaches. We now know that gene expression in the natural environments of *B. burgdorferi* , either a tick or a vertebrate, is immensely different from that in culture, despite many systems developed to mimic the in vivo situation (Grove et al. 2017; Iyer et al. 2015; Sapiro et al. 2023). A major strength of Grassmann et al. (2025) is the use of mammalian‐adapted spirochetes via an ingenious dialysis membrane chamber (DMC) system (Akins et al. 1998). They convincingly show that transcriptomes from host‐adapted spirochetes in DMCs are very different from transcriptomes from cultured bacteria across all genetic backgrounds and, in fact, transcriptomes from similar growth conditions cluster more tightly than transcriptomes from the same genotypes. Because BosR regulates production of RpoS, the RpoS‐independent BosR regulon was cleverly defined using an inducible *rpoS* mutant in which levels of the alternative sigma factor were dependent on IPTG and not BosR (Grassmann et al. 2025, 2023). The BosR regulon contained many expected genes required for vertebrate infection, such as *rpoS* and the canonical RpoS‐dependent genes (Caimano et al. 2019, 2007; Hübner et al. 2001), including *ospC*, which encodes an outer membrane lipoprotein crucial for establishing infection (Fingerle et al. 2007; Grimm et al. 2004; Pal et al. 2004), *dbpBA*, which encode decorin‐binding proteins (Fischer et al. 2003; Shi et al. 2008), and *bbk32*, which encodes a fibronectin‐binding protein (Fischer et al. 2006; Seshu et al. 2006), as well as chemotaxis genes. RpoS‐dependent BosR‐repressed genes also included several genes required for the tick phase, notably *ospAB*, which encode outer membrane lipoproteins needed in the tick (Battisti et al. 2008; Yang et al. 2004), as well as the glycerol utilization operon, which facilitates survival in the tick (Drecktrah et al. 2022; He et al. 2011; Pappas et al. 2011) and appears to be under the control of all known gene regulatory systems (Caimano et al. 2016; Samuels et al. 2021). The RpoS‐independent component of the BosR regulon showed fairly modest changes in gene expression and included mostly genes of unknown function as well as some genes involved in chromosome partitioning and a few lipoproteins: many of these BosR‐regulated and RpoS‐independent genes are carried on the cp32 plasmids, which are a suite of highly similar 32‐kb circular plasmids that are prophages capable of transduction (Eggers et al. 2001, Eggers and Samuels 1999, Faith et al. 2024). This suggests that BosR may provide a mechanistic bridge between RpoS‐mediated transmission from tick to vertebrate along with adaptation to the host environment and the putative phage‐generated diversity required for maintenance of the spirochete in its host population (Wachter et al. 2023). Absent from the in vivo transcriptomes were genes involved in the oxidative stress response and transition metal homeostasis; so, a lingering question in the field regarding the role of BosR has finally been satisfactorily settled.

In recent years, the extensive roles and structural diversity of RNA‐binding proteins (RBPs) have been recognized; they function in a variety of key processes in bacteria via several different mechanisms (Babitzke et al. 2019; Ng Kwan Lim et al. 2021). We discovered the first RBP in *B. burgdorferi* many years ago (Lybecker et al. 2010), which has recently been characterized as FlgV (Zamba‐Campero et al. 2024), a flagellar assembly protein that we hypothesize moonlights as an RNA chaperone (Van Gundy et al. 2025); we also showed that PlzA, the sole c‐di‐GMP target in the Lyme disease spirochete, is an RBP and has RNA chaperone activity in vitro (Van Gundy et al. 2023). A recent seminal study determined that BosR binds the *rpoS* mRNA and post‐transcriptionally regulates *rpoS* gene expression (Raghunandanan et al. 2024, 2025). Raghunandanan et al. (2024) used *rpoS* transcriptional and translational fusions along with RNA turnover analyses to show that BosR regulates *rpoS* expression by stabilizing and, thus, increasing the half‐life of the *rpoS* mRNA. Subsequently, they co‐immunoprecipitated *rpoS* mRNA with BosR to directly demonstrate in vivo RNA binding, which was further supported by in vitro RNA electrophoretic mobility shift assays (Raghunandanan et al. 2024). We also recently identified BosR as an RNA‐binding protein in a non‐biased approach that co‐sedimented BosR with small RNAs in a glycerol gradient and then demonstrated that BosR had in vitro RNA chaperone activity (Van Gundy et al. 2025). The BosR transcriptomes, which assay steady‐state RNA levels, cannot determine whether BosR is binding to DNA and regulating transcription or is binding to mRNA and regulating RNA stability (or, presumably, acting on both nucleic acids). Surely, BosR binds to promoter regions and affects RNA polymerase initiation; the transcriptome data likely reflect the influence of both RNA‐ and DNA‐binding activities of BosR. Unfortunately, Grassmann et al. (2025) discount the data on the RNA‐binding and post‐transcriptional regulatory activity of BosR and conclude that BosR lacks a consensus DNA‐binding site. Perhaps this is accurate, but the pool of sequences from the BosR regulon almost certainly includes a considerable amount of DNA that is not bound by BosR, which will confound the search analysis; frankly, one cannot define a DNA recognition site in vivo without a chromatin immunoprecipitation and deep sequencing type of experiment (ChIP‐seq). Moreover, to delineate the respective contributions of RNA‐ and DNA‐binding to the role of BosR in gene expression, the in vivo RNA interactome of BosR should be elucidated by crosslinking immunoprecipitation or RNA immunoprecipitation followed by high‐throughput sequencing (CLIP‐seq or RIP‐seq) under physiologically relevant conditions using the DMC model or, if possible, in ticks (Lybecker and Samuels 2017).

Overall, we have learned an immense amount about BosR, one of the most important regulators of gene expression in *B. burgdorferi* , from these recent studies regarding a new mechanism of regulation (Raghunandanan et al. 2024) and a carefully curated in vivo regulon (Grassmann et al. 2025). BosR most likely functions by binding both DNA and RNA; the relative contributions of these two activities to the physiology of the spirochete as it traverses its enzootic cycle will not be able to be molecularly dissected until BosR mutants are available that bind only one of the two nucleic acids, which may or may not be feasible to construct, but will require more structural data.

## Author Contributions

**D. Scott Samuels:** conceptualization, writing – original draft, writing – review and editing. **Meghan C. Lybecker:** writing – original draft, writing – review and editing.

## Funding

This work was supported by National Institutes of Health grants U01 AI169840‐01A1 (to C. Davies, R.T. Marconi, and DSS) and R01 AI173045‐01 (to P.R. Secor with DSS). [Correction added on 14 January 2026 after first online publication: The grant number in the funding has been corrected.]

## Linked Articles

This article is linked to Grassmann et al. papers. To view this article, visit https://doi.org/10.1111/mmi.70036.

## Data Availability

Data sharing not applicable to this article as no datasets were generated or analysed during the current study.
